# Change in resuscitation influenced development and severity of inflammatory complications in severely injured

**DOI:** 10.1007/s00068-025-02905-8

**Published:** 2025-06-23

**Authors:** Karlijn J. P. van Wessem, Kim E. M. Benders, Luke P. H. Leenen, Falco Hietbrink

**Affiliations:** Department of Trauma Surgery, University Medical Center Utrecht, Suite G04.232, Heidelberglaan 100, Utrecht, 3584 CX The Netherlands

**Keywords:** Resuscitation, Crystalloids, FFP, Inflammatory complications, Mortality, Polytrauma

## Abstract

**Introduction:**

Resuscitation strategies for severely injured patients have shifted toward reduced crystalloids and increased balanced blood product resuscitation, including Fresh Frozen Plasma (FFP) to reduce organ failure and mortality. However, FFP is associated with higher infection and sepsis risks. This study investigated the impact of resuscitation changes on inflammatory complications and mortality.

**Methods:**

This 11-year cohort study included severely injured patients (> 15 years) admitted to a Level-1 Trauma Center ICU. Exclusions included isolated head injuries, drowning, asphyxiation, burns, and deaths < 48 h. Data on demographics, resuscitation, inflammatory complications (MODS, ARDS, infections, thromboembolism), and mortality were collected.

**Results:**

Among 585 patients (median age 46,72% male, ISS 29, 94% blunt injuries), 18% developed MODS, 3% ARDS, 45% infections, 9% thromboembolism, and 14% died. Over time, crystalloids ≤ 24 h decreased while FFP ≤ 24 h increased, correlating with reduced ARDS but increased thromboembolic events. Crystalloids ≤ 24 h independently predicted MODS, infections, and mortality, while FFP ≤ 24 h was linked to MODS and thromboembolism. Causes of death other than neurological included MODS (5%), sepsis (3%), and ARDS (1%), with no deaths from thromboembolic complications.

**Conclusion:**

Resuscitation evolved toward less crystalloids and more FFP ≤ 24 h, likely reducing ARDS but increasing thromboembolic complications, while other outcomes remained comparable. Low mortality from inflammatory complications suggests these complications were mild. The anti-inflammatory, immune-modulating effect of FFP might have played a role in the attenuation of these complications, supporting current resuscitation strategies. However, improved identification of patients who require FFPs may help reduce thromboembolism. In the future, optimal FFP dosage should be determined to balance coagulopathy correction, blood volume restoration, and management of the inflammatory response following trauma.

**Supplementary Information:**

The online version contains supplementary material available at 10.1007/s00068-025-02905-8.

## Introduction

Advances in trauma care have attributed to better survival and a change in cause of death. Strategies in early hemorrhage control and changes in hemostatic resuscitation have decreased death by exsanguination, and traumatic brain injury (TBI) related deaths have become the biggest challenge in trauma care [1, 2].

Classically, severe trauma is associated with blood loss resulting in hypoperfusion and acidosis. The systemic inflammatory response that is initiated by trauma causes the endothelium to be activated which becomes porous causing tissue edema. As a consequence, patients require volume suppletion to maintain an adequate tissue perfusion and many patients also develop coagulopathy. In order to supplement this volume depletion crystalloid resuscitation has been advocated. However, in the early 2000s, it became clear that liberal crystalloid resuscitation was associated with increased inflammatory complications such as Multiple Organ Dysfunction Syndrome (MODS) and Adult Respiratory Distress Syndrome (ARDS). In order to decrease these complications, high volume crystalloid resuscitation was abandoned and damage control resuscitation (DCR) was introduced in order to break the triad of acidosis, hypothermia and coagulopathy by quickly treating the cause of bleeding, reducing hypothermia, and correction of blood volume and coagulopathy by administration of blood without large volumes of crystalloids [3, 4]. This strategy has not only reduced mortality rates in the past decade, but also influenced the cause of death. Death by exsanguination, MODS-, and ARDS-related deaths decreased, whereas TBI became one of the largest causes of death [5, 6].

In order to investigate the influence of resuscitation changes in the first 24 h after trauma on the development of inflammatory complications such as MODS, ARDS, infectious and thromboembolic complications, we conducted a retrospective analysis of prospectively collected data in a cohort of severely injured patients. We hypothesized that during the studied period a change in resuscitation strategies could be observed and that this change would have an influence on the development and severity of inflammatory complications and thus outcomes.

## Methods

### Study setting

An 11-year retrospective analysis of prospectively collected data from trauma patients treated at a major urban Level-1 trauma center was conducted. In 2014, a prospective population-based cohort study started, enrolling all consecutive trauma patients admitted to the Intensive Care Unit (ICU). Detailed descriptions of the hospital and its catchment area have been published previously [7]. Included patients were admitted to the ICU either directly from the emergency department (ED) or postoperatively following emergency surgery. Exclusion criteria included patients under 16 years old, those with isolated TBI (defined as an Abbreviated Injury Score (AIS) of 3 or higher for the head and 2 or lower in other regions), asphyxiation, drowning, or burns, as these conditions have distinct physiological responses and significantly different morbidity and mortality profiles [8, 9]. Additionally, patients who died within 48 h were excluded. A flowchart illustrating patient selection is available in Supplemental Figure S1.

### Data collection

Data were recorded prospectively upon ED arrival and daily in the ICU by authors KW and LL. Collected parameters included patient demographics, Injury Severity Score (ISS), and indicators of shock and resuscitation. Blood product usage—including Packed Red Blood Cells (PRBC), Fresh Frozen Plasma (FFP), and Platelets (PLT)—was documented within the first 24 h of admission. Blood transfusion indications followed the hospital’s transfusion protocol, implemented in 2013, and included (massive) hemorrhage, coagulopathy (prothrombin time > 1.5× normal values), and damage control resuscitation with a 1:1:1 blood component ratio. In 2018, initial crystalloid administration was reduced from 2 L to 1 L in accordance with the 10th edition of the Advanced Trauma Life Support (ATLS) guidelines [10]. Thromboelastography was not routinely used.

### Multiple organ dysfunction syndrome (MODS)/adult respiratory distress syndrome (ARDS)

Daily documentation continued for up to 28 days or until ICU discharge, including Denver Multiple Organ Failure (MOF) scores [11] and ARDS Berlin criteria [12]. The Denver MOF score was preferred over other scoring systems (Marshall MODS, SOFA) due to the exclusion of the Glasgow Coma Scale (GCS), which can be unreliable in ICU trauma patients who are frequently sedated and intubated [9].

### Thrombosis prophylaxis and thromboembolic complications

All patients received Low Molecular Weight Heparin (LMWH) for thrombosis prophylaxis within 24 h unless contraindicated by active bleeding, recent major surgery, or severe TBI with a high risk of exacerbating intracranial bleeding. In cases where early anticoagulation was not feasible, graduated compression stockings were used when applicable (i.e., no lower leg casts or external fixators). Prophylactic vena cava filters were not routinely used.

In-hospital arterial and venous thromboembolic events, including pulmonary embolism (PE), deep vein thrombosis (DVT), and arterial thrombosis (e.g., myocardial infarction, stroke), were recorded. PE was diagnosed on CT and included all types of pulmonary embolism, including (sub)segmental PEs if treatment was started based on the CT images. DVT was diagnosed by ultrasound or CT, and arterial thrombosis based on clinical presentation and imaging. There is no routine screening protocol for thromboembolic complications in our hospital.

### Infectious complications

Infections were classified using criteria adapted from the Centers for Disease Control and Prevention (CDC) [13], American College of Surgeons Wound Schema [14], Barts Health NHS Trust guidelines [15], and consensus definitions for specific infections [16, 17]. Full definitions are available in Supplemental Table S2.

The primary outcome was the relationship between early resuscitation (first 24 h) and the incidence of inflammatory complications, including MODS, ARDS, infections, and thromboembolic events during hospitalization.

### Ethical approval

The study received ethical approval from the local ethics committee (Reference number: WAG/mb/16/026664).

### Statistical analysis

Data were analyzed using IBM SPSS Statistics, version 30.0 (Armonk, NY, USA). Graphs were prepared with GraphPad Prism version 10.3.0 (San Diego, CA, USA). Results were presented as median with interquartile range (IQR). Crystalloids and blood products were divided into 4 predefined, clinically relevant, volume groups for easier comparison. Kruskal-Wallis test or Pearson-Chi-square test in dichotomous data were used to compare variables. A multivariate analysis was performed by forward stepwise logistic regression with the separate inflammatory complications and mortality as the dependent variable to identify independent risk factors for the development of posttraumatic inflammatory complications and mortality. The variables that were analyzed as potential predictors were selected from literature and from our clinical experience. These variables consisted of patient characteristics (age, gender), severity and type of injury (ISS, pelvic fracture), early physiology (systolic blood pressure, blood gas), and early treatment (crystalloid and blood transfusion, tranexamic acid, urgent laparotomy). Data were presented as odds ratios and 95% confidence interval. Statistical significance was defined as *P* < 0.05.

## Results

During the 11-year study period 585 patients who survived the first 48 h were included. 72% were male with a median age of 46 (28–61) years. 94% sustained blunt injury with a median ISS of 29 (22–38). The demographics and outcome parameters are shown in Table 1. Since the overall administration of platelets (PLT) ≤ 24 h was very low, they were not included in further analysis.

**Table 1:** Demographics

Demographics	Total population (*n* = 585)
Age (years)	46 (28–61)
Male gender	423 (72)
Blunt MOI	549 (94)
ISS	29 (22–38)
AIS head	3 (1–4)
AIS face	0 (0–2)
AIS chest	3 (2–4)
AIS abdomen	2 (0–3)
AIS pelvis/extremities	2 (0–3)
AIS external	1 (0–1)
Pelvic fracture	187 (32)
Urgent laparotomy (≤ 24 h)	144 (25)
SBP_ED (mmHg)	120 (96–140)
SBP ≤ 90 mmHg_ED	126 (22%)
Hb-ED (mmol/L)	8.1 (7.4–8.9)
BD _ED (mEq/L)	−3.0 (−6.0- −0.8)
PT_ED (sec)	13.8 (12.5–15.6)
SBP _ICU (mmHg)	119 (105–135)
Hb_ICU (mmol/L)	7.6 (6.9–8.4)
BD_ICU (mEq/L)	−4.0 (−6.1- −2.0)
Resuscitation parameters	
Crystalloids ≤ 24 h (L)	6.7 (4.5–9.4)
PRBC ≤ 24 h (U)	2 (0–6)
PRBC ≥ 10 units ≤ 24 h	68 (12)
FFP ≤ 24 h (U)	2 (0–6)
FFP ≥ 10 units ≤ 24 h	86 (15)
PLT ≤ 24 h (U)^#^	0 (0–1)
TXA ≤ 24 h	394 (67)
Outcome parameters	
Ventilator days	5 (2–11)
ICU LOS (days)	7 (3–13)
H-LOS (days)	21 (12–33)
MODS	103 (18)
ARDS	18 (3)
Infectious complications	262 (45)
Thromboembolic complications	52 (9)
Mortality	79 (14)

Data are expressed in median (IQR) or absolute numbers (%)

^#^1 unit of platelets contains 5 donors

MOI = mechanism of injury, ISS = Injury Severity Score, AIS = Abbreviated Injury Scale, SBP = systolic blood pressure, ED = Emergency Department, Hb = hemoglobin, BD = Base Deficit, PT = prothrombin time, PRBC = Packed Red Blood Cells, FFP = Fresh Frozen Plasma, PLT = Platelets, TXA = tranexamic acid, MODS = Multiple Organ Dysfunction Syndrome, ARDS = Adult Respiratory Distress Syndrome, ICU = Intensive Care Unit, LOS = length of stay, H-LOS = hospital length of stay

In this study 18% of patients developed MODS, 3% ARDS, 45% infectious complications, and 9% thromboembolic complications. Mortality was 14%, the vast majority died of TBI (72%). Death due to MODS was 5% (*n* = 4), sepsis 3% (*n* = 2), and ARDS 1% (*n* = 1). Other causes of death were respiratory failure (10%), cardiac origin (4%), ischemia (1%), hypoxia (1%) and other miscellaneous causes (2%).

### Relation between injury severity, resuscitation and inflammatory complications and mortality

**Fig. 1:**
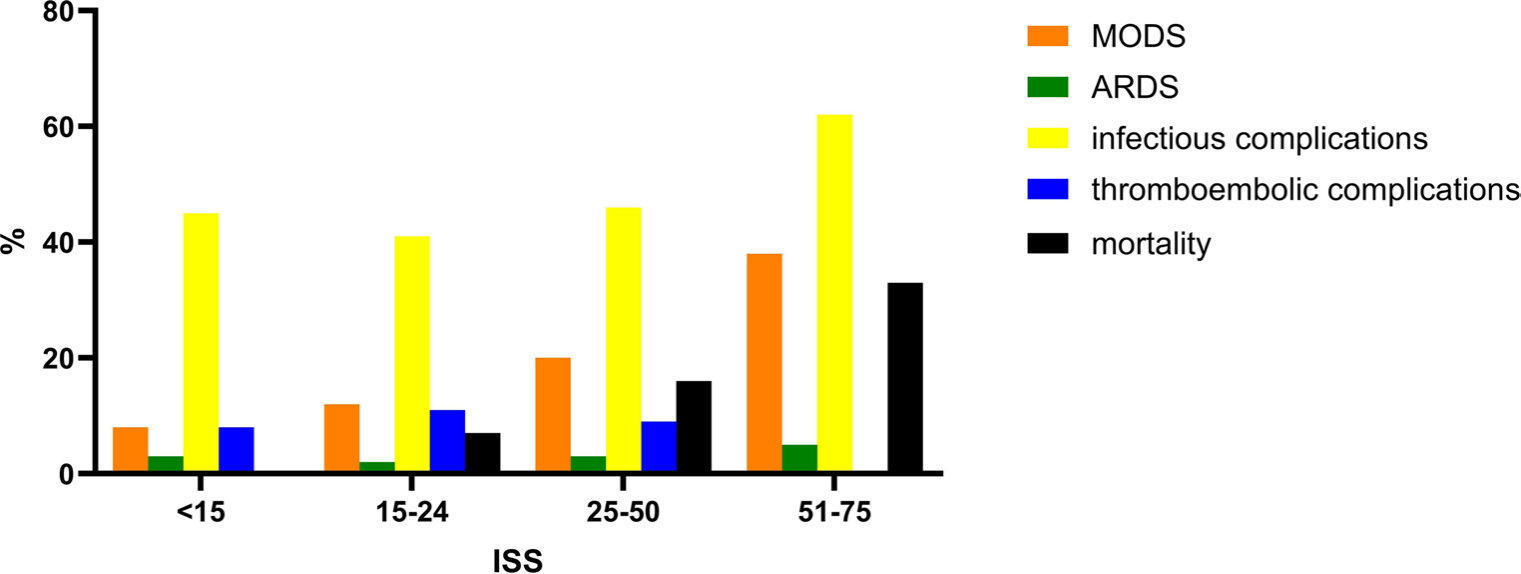
Injury severity (ISS) related to inflammatory complications and mortality

MODS and mortality increased significantly (*p* = 0.004 and *p* < 0.001, respectively) with increasing injury severity whereas ARDS (*p* = 0.83), infectious (*p* = 0.33) and thromboembolic complications (*p* = 0.39) were comparable between the ISS groups (Fig. 1).

The relation between clinically relevant volume groups of crystalloids and blood products ≤ 24 h and the development of inflammatory complications and mortality is depicted in Table 2. All inflammatory complications and mortality increased with increasing crystalloids ≤ 24 h. With increasing PRBC ≤ 24 h and FFP ≤ 24 h MODS, infectious and thromboembolic complications, and mortality increased, while ARDS remained low in all volume groups.

**Table 2:** Distribution of outcome across different volume groups of crystalloids, PRBC, and FFP

	MODS (*n* = 103)	ARDS (*n* = 18)	Infectious complications (*n* = 262)	Thromboembolic complications (*n* = 52)	Mortality (*n* = 79)
**Crystalloids**					
0–5 L (*n* = 178)	18 (10)	0	61 (34)	10 (6)	17 (10)
5–10 L (*n* = 286)	51 (18)	13 (5)	141 (49)	27(9)	33 (12)
10–15 L (*n* = 100)	26 (26)	3 (3)	47 (47)	11 (11)	23 (23)
≥ 15 L (*n* = 21)	8 (38)	2 (10)	13 (62)	4 (19)	6 (29)
P-value	< 0.001*	0.01*	0.003*	0.02*	< 0.001*
**PRBC**					
0 U (*n* = 249)	29 (12)	8 (3)	95 (38)	13 (5)	27 (11)
1–4 U (*n* = 157)	20 (13)	3 (2)	75 (48)	15 (10)	20 (13)
5–9 U (*n* = 111)	32 (29)	4 (4)	54 (49)	14 (13)	17 (15)
≥ 10 U (*n* = 68)	22 (32)	3 (4)	38 (67)	10 (15)	15 (22)
P-value	< 0.001*	0.64	0.004*	0.003*	0.02*
**FFP**					
0 U (*n* = 271)	27 (10)	6 (2)	108 (40)	13 (5)	30 (11)
1–4 U (*n* = 126)	23 (18)	7 (6)	57 (45)	13 (10)	15 (12)
5–9 U (*n* = 102)	24 (23)	3 (3)	48 (47)	11 (11)	16 (16)
≥ 10 U (*n* = 86)	29 (34)	2 (2)	49 (57)	15 (17)	18 (21)
P-value	< 0.001*	0.85	0.006*	< 0.001*	0.02*

Data are expressed in absolute numbers (%), *statistically significant

PRBC = Packed Red Blood Cells, FFP = Fresh Frozen Plasma, MODS = Multiple Organ Dysfunction Syndrome, ARDS = Adult Respiratory Distress Syndrome, U = units

### Trends in time

**Fig. 2:**
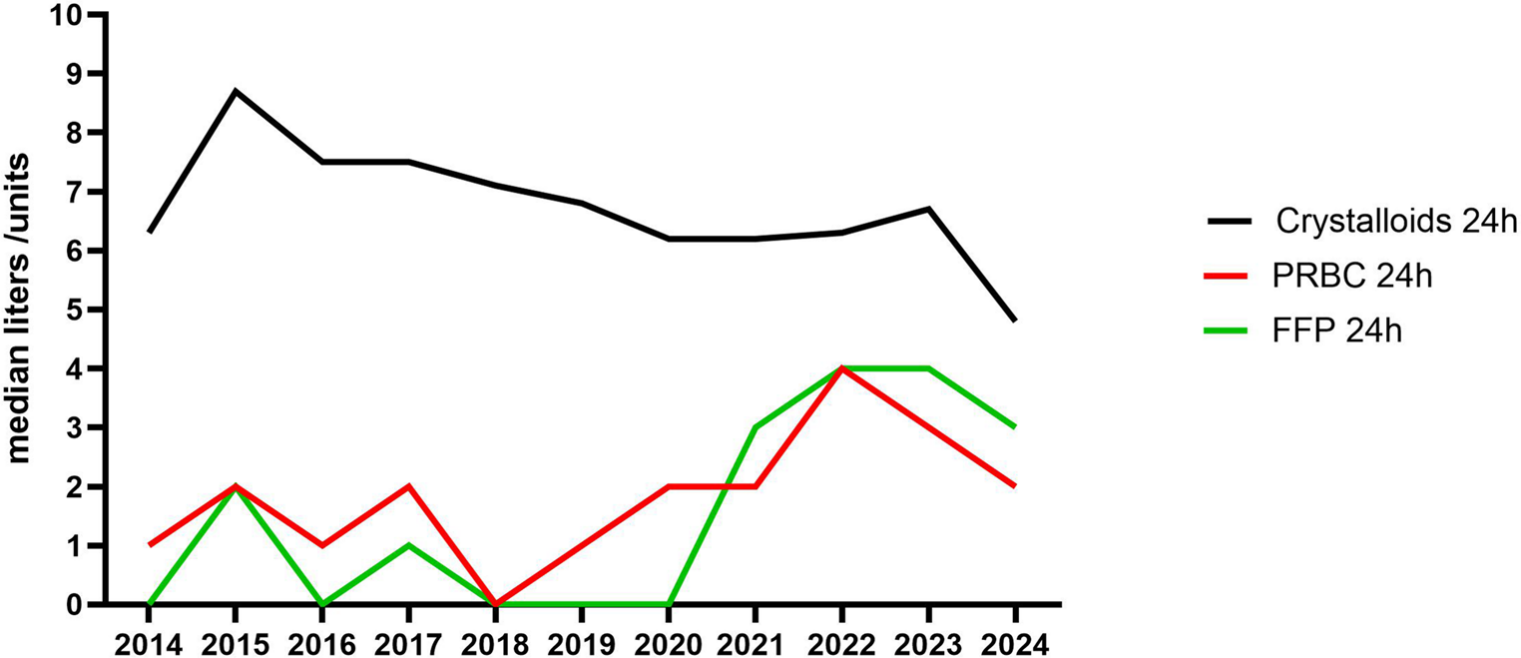
Resuscitation over time: Crystalloids, packed red blood cells (PRBC), and Fresh Frozen Plasma (FFP) ≤ 24 h after admission

There were no differences in demographics and injury severity over time, although the number of pelvic fractures increased, mainly in the last 3 years. No changes were observed in physiology except for prothrombin time in ED (PT_ED) and base deficit in ICU (BD_ICU) that decreased, whereas hemoglobin in ICU (Hb_ICU) increased over time (Table 3). In the 11-year study period the amount of administered crystalloids ≤ 24 h decreased (*p* < 0.001), whereas the number of administered FFP ≤ 24 h increased (Table 3; Fig. 2, *p* < 0.001). There was no difference in PRBC ≤ 24 h administration (*p* = 0.68, Table 3; Fig. 2).

**Table 3:** Demographics, physiology, resuscitation and outcome per studied year

	2014 (*n* = 43)	2015 (*n* = 46)	2016 (*n* = 62)	2017 (*n* = 56)	2018 (*n* = 60)	2019 (*n* = 65)	2020 (*n* = 65)	2021 (*n* = 61)	2022 (*n* = 48)	2023 (*n* = 38)	2024 (*n* = 41)	*P*-Value
Age (years)	40 (23–58)	39 (25–56)	49 (33–66)	52 (31–60)	44 (26–57)	51 (32–70)	35 (26–54)	47 (31–58)	39 (29–61)	48 (30–57)	50 (35–63)	0.35
Male gender (%)	34 (79)	38 (83)	42 (68)	44 (79)	41 (68)	37 (57)	45 (69)	43 (70)	37 (77)	32 (84)	30 (73)	0.82
Blunt MOI (%)	41 (95)	45 (98)	60 (97)	53 (95)	59 (98)	61 (95)	60 (92)	52 (85)	43 (90)	36 (95)	39 (95)	0.05
ISS	30 (25–43)	29 (22–37)	29 (20–34)	29 (23–36)	27 (22–34)	27 (22–34)	29 (23–38)	29 (22–37)	33 (25–40)	30 (27–45)	29 (22–39)	0.25
Pelvic fracture (%)	8 (19)	13 (28)	17 (27)	23 (41)	22 (37)	18 (28)	15 (23)	15 (25)	20 (42)	19 (50)	17 (42)	0.02*
Urgent laparotomy (%)	9 (21)	14 (30)	8 (13)	20 (36)	14 (23)	17 (26)	12 (18)	21 (34)	8 (17)	9 (24)	12 (29)	0.70
SBP_ED ≤ 90 mmHg (%)	8 (19)	10 (22)	12 (19)	13 (23)	11 (18)	12 (18)	8 (12)	18 (30)	14 (29)	9 (24)	11(27)	0.19
Hb_ED (mmol/L)	8.2 (7.3–8.9)	8.0 (7.2–8.8)	7.8 (7.2–8.9)	8.3 (7.4–9.2)	8.1 (7.4–9.1)	8.0 (7.2–8.8)	8.2 (7.5–9.2)	8.3 (7.7–9.0)	8.2 (7.6–8.7)	8.5 (7.4–9.1)	7.9 (7.0–8.8)	0.33
BD_ED (mEQ/L)	−3.0 (−6.0-0.0)	−3.0 (−6.0—1.0)	−2.8 (−6.0—0.8)	−3.0 (−5.0-0.0)	−2.0 (−6.0-1.0)	−3.0 (−6.0–0.5)	−3.0 (−4.5—0.5)	−4.0 (−8.0—0.5)	−4.0 (−7.0—2.0)	−4.0 (−7.0—1.0)	−3.0 (−6.8—2.0)	0.08
PT_ED (sec)	16.3 (14.6–17.9)	16.6 (15.1–18.9)	15.8 (14.4–18.0)	15.5 (14.3–17.2)	14.5 (13.0–15.6)	12.7 (12.1–13.4)	13.1 (12.0–13.7)	13.0 (12.1–13.8)	12.9 (11.6–14.1)	12.6 (11.7–14.4)	12.2 (11.6–13.3)	< 0.001*
Hb_ICU (mmol/L)	7.1 (6.5–7.9)	7.3 (6.5–7.8)	7.4 (6.6–8.3)	7.9 (6.9–8.4)	7.8 (7.0–8.4)	7.7 (7.0–8.3)	7.8 (7.0–8.6)	8.0 (7.2–8.8)	7.6 (7.0–8.2)	8.0 (7.2–8.6)	7.6 (6.9–8.2)	0.001*
BD_ICU (mEQ/L)	−2.5 (−5.0—0.8)	−3.5 (−5.0—0.6)	−3.1 (−5.2—1.9)	−3.5 (−5.7—1.4)	−4.3 (−6.9—2.1)	−4.6 (−6.8—2.3)	−4.2 (−6.8—2.3)	−4.6 (−6.9—3.2)	−4.3 (−6.7—3.0)	−4.5 (−6.1 − 2.6)	−4.6 (−6.0—2.4)	< 0.001*
Crystalloids ≤ 24 h (L)	6.3 (4.5–10.0)	8.7 (5.8–11.5)	7.5 (4.9–10.5)	7.5 (5.1–10.4)	7.1 (4.4–8.6)	6.8 (4.6–10.1)	6.2 (3.5–9.0)	6.2 (4.3–8.2)	6.3 (4.5–8.3)	6.7 (4.7–9.4)	4.8 (3.3–7.0)	< 0.001*
PRBC ≤ 24 h (U)	1 (0–5)	2 (0–7)	1 (0–5)	2 (0–6)	0 (0–4)	1 (0–5)	2 (0–5)	2 (0–7)	4 (0–8)	3 (0–7)	2 (0–8)	0.68
PRBC ≥ 10 ≤ 24 h(%)	4 (9)	8 (17)	8 (13)	4 (7)	3 (5)	7 (11)	8 (12)	9 (15)	9 (19)	4 (11)	4 (10)	0.66
FFP ≤ 24 h (U)	0 (0–4)	2 (0–6)	0 (0–5)	1 (0–6)	0 (0–4)	0 (0–6)	0 (0–6)	3 (0–9)	4 (0–11)	4 (0–9)	3 (0–8)	0.01*
TXA ≤ 24 h	22 (51)	30 (68)	43 (69)	37 (66)	37 (62)	44 (68)	45 (69)	43 (70)	34 (71)	26 (68)	33 (80)	0.03*
Ventilator days	7 (3–14)	7 (2–12)	7 (4–12)	7 (2–12)	6 (2–11)	5 (2–10)	4 (2–10)	4 (2–7)	6 (2–11)	7 (4–18)	4 (2–7)	0.06
Ventilator free days	10 (5–15)	16 (10–20)	13(6–16)	11(3–19)	11 (0–16)	13(4–20)	14 (7–21)	12 (7–23)	10 (2–26)	15 (7–29)	18 (5–28)	0.02*
ICU_LOS (days)	8 (3–15)	7 (4–14)	10 (5–14)	7 (3–16)	6 (3–12)	7 (3–13)	6 (3–12)	5 (3–10)	7 (3–12)	8 (5–20)	5 (2–11)	0.05
H_LOS (days)	20 (11–37)	29 (19–39)	22 (14–31)	23 (12–34)	17 (10–28)	19 (9–29)	20 (13–31)	16 (11–31)	20 (10–35)	26 (15–42)	22 (10–38)	0.58
ARDS (%)	7 (16)	2 (4)	3 (5)	2 (4)	1 (2)	1(2)	0	1(2)	0	0	1 (2)	< 0.001*
MODS (%)	9 (21)	2 (4)	12(19)	14 (25)	10 (17)	9 (14)	10 (15)	7 (11)	11 (23)	10 (26)	9 (22)	0.32
Infectious complications (%)	16 (37)	14 (30)	33 (53)	30 (54)	25 (42)	26 (40)	31 (48)	25 (41)	26 (54)	22 (58)	14 (34)	0.43
Thromboembolic complications (%)	0	2 (4)	3 (5)	6 (11)	1 (2)	6 (9)	11 (17)	8 (13)	4 (8)	4 (11)	7 (17)	0.001*
Mortality (%)	6 (14)	4 (9)	10 (16)	7 (13)	8 (13)	11 (17)	9 (14)	8 (13)	7 (15)	5 (13)	4 (10)	0.91

Data are expressed as median(IQR) or absolute numbers (%)

*statistically significant

MOI = mechanism of injury, ISS = injury severity score, AIS = abbreviated injury scale, SBP = systolic blood pressure, ED = emergency department, Hb = hemoglobin, BD = base deficit, PT = prothrombin time, ICU = intensive care unit, LOS = length of stay, H_LOS = length of stay in hospital, TXA = tranexamic acid, MODS = multiple organ dysfunction syndrome, ARDS = adult respiratory distress syndrome

**Fig. 3:**
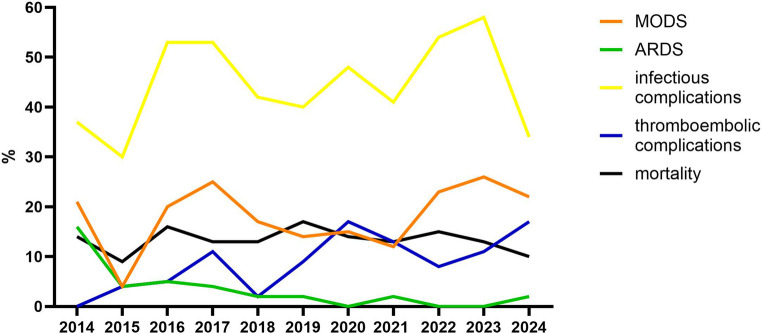
Inflammatory complications and mortality over time

The incidence of ARDS decreased (*p* < 0.001), and thromboembolic complication rates (*p* < 0.001) increased during the study period. MODS (*p* = 0.32), infectious complications (*p* = 0.43), and mortality (*p* = 0.91) did not change over time (Table 3; Fig. 3).

### Injury severity and physiology related to FFP administration

Since the administration of FFPs increased during the studied period, the role of FFP on the development of inflammatory complications was investigated more into detail; the distribution of injury severity, physiology in ED and ICU across the different FFP volume groups is displayed in Table 4. Patients who received more FFPs were younger than patients who received less or no FFPs. An increase in injury severity, and a more deranged physiology both in ED and ICU was associated with more FFP ≤ 24 h. More FFP ≤ 24 h were accompanied by more PRBC ≤ 24 h, more PLT ≤ 24 h, and more frequently administered tranexamic acid (TXA) in the first 24 h. Additionally, patients who received more FFPs stayed longer on the ventilator, longer in ICU, longer in hospital, and died more often. Patients who received more FFPs developed more often MODS, infectious and thromboembolic complications (Table 2).

**Table 4:** Distribution of injury severity and physiology across different FFP volume groups

	0 FFPs (*n* = 271)	1–4 U FFPs (*n* = 126)	5–9 U FFPs (*n* = 102)	10 + FFPs (*n* = 86)	*P*-value
Age (years)	49 (29–63)	43 (30–58)	41 (25–55)	38 (25–63)	0.03*
Male gender	188 (70)	93 (74)	79 (77)	63 (73)	0.22
ISS	28 (21–34)	29 (23–38)	34 (25–41)	34 (26–43)	< 0.001*
Urgent laparotomy (≤ 24 h)	12 (4)	34 (27)	47 (46)	51 (59)	< 0.001*
SBP_ED (mmHg)	132 (114–150)	120 (101–136)	108 (88–130)	83 (70–105)	< 0.001*
Hb_ED (mmol/L)	8.4 (7.8–9.1)	8.2 (7.4–8.9)	7.7 (7.0–8.5)	7.4 (6.5–8.4)	< 0.001*
pH_ED	7.33 (7.28–7.38)	7.30 (7.24–7.36)	7.29 (7.23–7.36)	7.25 (7.10–7.32)	< 0.001*
BD_ED (mEq/L)	−2.0 (−4.0-1.0)	−4.0 (−6.0–2.0)	−5.0 (−7.5–2.0)	−8.0 (−13.0–4.0)	< 0.001*
PT_ED (Sec)	13.4 (12.1–15.1)	14.2 (12.5–16.8)	14.1 (13.2–16.8)	14.5 (13.3–17.7)	< 0.001*
SBP_ICU	122 (110–137)	120 (103–136)	114 (102–129)	110 (100–131)	0.01*
Hb_ICU (mmol/L)	7.9 (7.2–8.5)	7.5 (6.8–8.2)	7.3 (6.5–8.0)	7.5 (6.7–8.2)	< 0.001*
pH_ICU	7.34 (7.30–7.38)	7.33 (7.27–7.37)	7.34 (7.29–7.38)	7.32 (7.26–7.38)	0.03*
BD_ICU (mEq/L)	−3.4 (−5.3—1.5)	−4.8 (−6.4—2.2)	−4.2 (−6.2—2.6)	−5.5 (−7.9—3.5)	< 0.001*
Crystalloids ≤ 24 h (L)	5.2 (3.3–7.2)	7.7 (5.1–10.8)	8.4 (6.6–10.7)	8.9 (6.7–11.4)	< 0.001*
PRBC ≤ 24 h (U)	0 (0–0)	3 (1–4)	6 (4–8)	12 (8–15)	< 0.001*
PLT ≤ 24 h (U)^#^	0 (0–0)	0 (0–1)	1 (0–2)	2 (1–3)	< 0.001*
TXA ≤ 24 h	135 (50)	92 (73)	87 (85)	80 (93)	< 0.001*
Ventilator days	5 (2–9)	5 (2–11)	6 (3–11)	7 (4–14)	0.005*
Ventilator free days	12 (5–18)	13 (6–20)	13 (7–21)	15 (1–24)	0.02*
ICU_LOS (days)	6 (3–12)	7 (3–14)	8 (4–13)	8 (5–16)	0.01*
H_LOS (days)	18 (11–30)	22 (14–34)	24 (14–37)	25 (13–39)	< 0.001*

Data are expressed in median (IQR) or absolute numbers (%)

MOI = mechanism of injury, ISS = Injury Severity Score, SBP = systolic blood pressure, ED = Emergency Department, Hb = hemoglobin, BD = Base Deficit, PT = prothrombin time, PRBC = Packed Red Blood Cells, FFP = Fresh Frozen Plasma, PLT = Platelets, TXA = tranexamic acid, ICU = Intensive Care Unit, LOS = length of stay, H-LOS = hospital length of stay

All 4 patients who died of MODS had ≥ 10 FFP ≤ 24 h. The patient who died of ARDS did not receive any FFPs, and 1 of the patients who died of sepsis had between 1 and 4 FFP ≤ 24 h and the other one received ≥ 10 FFP ≤ 24 h. No patients died of thromboembolic complications.

### Independent predictors for development of inflammatory complications and mortality

Multivariate analysis with stepwise forward selection was performed to identify independent predictors for the development of the different inflammatory complications and mortality. Based on separate univariate analysis per inflammatory complication or mortality parameters were chosen. Since the prevalence of ARDS was very low (*n* = 18) it was decided to exclude ARDS from further multivariate analysis. The parameters that were included for MODS were age, ISS, pelvic fracture, BD_ED, PT_ED, BD_ICU, crystalloids ≤ 24 h, PRBC ≤ 24 h and FFP ≤ 24 h. Age, ISS, crystalloids ≤ 24 and FFP ≤ 24 were shown to be independent predictors for MODS. Parameters that were included for infectious complications were age, ISS, pelvic fracture, crystalloids ≤ 24 h, PRBC ≤ 24 h and FFP ≤ 24 h. Age, pelvic fracture, and crystalloids ≤ 24 were shown to be independent predictors for infectious complications. Parameters that were included for thromboembolic complications were urgent laparotomy, crystalloids ≤ 24 h, PRBC ≤ 24 h, FFP ≤ 24 h, and TXA ≤ 24 h. Urgent laparotomy and FFP ≤ 24 were shown to be independent predictors for thromboembolic complications. Parameters that were included for mortality were age, ISS, BD_ED, BD_ICU, crystalloids ≤ 24 h, PRBC ≤ 24 h and FFP ≤ 24 h. Age, ISS, BD_ICU, and crystalloids ≤ 24 were shown to be independent predictors for mortality (Table 5).

**Table 5:** Multivariate analysis with Stepwise forward selection to identify independent predictors for MODS, infectious complications, thromboembolic complications and mortality

MODS	βcoefficient	*P*-value	Odds Ratio	95% C.I.	
				Lower	Upper
FFP ≤ 24 h	0.052	0.003	1.053	1.018	1.089
ISS	0.036	< 0.001	1.037	1.016	1.058
Age	0.016	0.011	1.016	1.004	1.029
Crystalloids ≤ 24 h	0.000	0.014	1.000	1.000	1.000
Constant	−4.445	< 0.001	0.012		
*Included parameters; step 1:FFP ≤ 24 h, step 2: ISS, step 3: age, and step 4: crystalloids ≤ 24 h*					
**Infectious complications**					
Pelvic fracture	0.569	0.002	1.766	1.236	2.525
Crystalloids ≤ 24 h	0.000	0.013	1.000	1.000	1.000
Age	0.010	0.030	1.010	1.001	1.018
Constant	−1.228	< 0.001	0.293		
*Included parameters; step 1:pelvic fracture, step 2: crystalloids ≤ 24 h, and step 3: age*					
**Thromboembolic complications**					
FFP ≤ 24 h	0.041	0.023	1.042	1.006	1.079
Urgent laparotomy	0.957	0.004	2.605	1.364	4.974
Constant	−2.884	< 0.001	0.056		
*Included parameters; step 1:FFP ≤ 24 h, and step 2: urgent laparotomy*					
**Mortality**					
Age	0.056	< 0.001	1.058	1.041	1.076
ISS	0.064	< 0.001	1.066	1.041	1.092
BD_ICU	−0.014	< 0.001	0.986	0.979	0.994
Crystalloids ≤ 24 h	0.000	0.00.1	1.000	1.000	1.000
Constant	−8.584	< 0.001	0.000		
*Included parameters; step 1:age, step 2: ISS, step 3: BD_ICU, and step 4: crystalloids ≤ 24 h*					

BD = Base Deficit, ED = Emergency Department, FFP = Fresh Frozen Plasma, ICU = Intensive Care Unit, ISS = Injury Severity Score, MODS = Multiple Organ Dysfunction Syndrome

## Discussion

In this 11-year study of severely injured patients admitted to ICU, early resuscitation shifted toward decreased crystalloids ≤ 24 h and increased FFP ≤ 24 h administration. Patients who were more severely injured, had a more deranged physiology, and who needed an urgent laparotomy more often received more FFP ≤ 24 h. Increased injury severity was associated with higher MODS and mortality rates, and increased crystalloids and blood administration correlated with higher rates of all inflammatory complications and mortality. Over time, ARDS incidence dropped, whereas thromboembolic complications increased, and rates of MODS, infectious complications, and mortality remained stable. Crystalloids ≤ 24 h independently predicted MODS, infectious complications, and mortality, while FFP ≤ 24 h was an independent predictor of MODS and thromboembolic complications.

Despite the association of FFP with inflammatory complications, mortality due to these complications was very low (1%), suggesting a milder clinical course. The reduction in ARDS is likely attributable to decreased crystalloid use, which is supported by previous research [18]. While FFP was historically associated with higher risks of MODS, ARDS, and infections [19–21], it may also have protective effects on the inflammatory response. Experimental studies suggest that FFP stabilizes the endothelial glycocalyx by inhibiting syndecan-1 shedding, reducing vascular permeability and the need for excessive fluid resuscitation [22, 23]. Additionally, FFP contains fibrinogen, which has anti-inflammatory and immune-modulating properties by inhibiting leukocyte recruitment [24]. These mechanisms may explain the observed milder inflammatory response despite increased FFP usage.

Age and injury severity scores did not change over the past decade in our polytrauma population, although there was an increase in pelvic fractures. Interestingly, BD_ICU decreased over the years indicating worse physiological conditions on arrival in ICU since base deficit is a result of the release of metabolites due to anaerobic metabolism triggered by hypoperfusion [25]. This implies that those patients had a higher likelihood of developing severe inflammatory complications. Also, BD_ICU was an independent predictor for mortality in this study. Nevertheless, this expected increase in inflammatory complications and death was not observed. Again, the influence of the anti-inflammatory, immune-modulating effect of FFP may have played a role in the attenuation of these complications.

It should be noted that PRBC administration remained stable over time while FFP increased. This was probably caused by the fact that most patients, although severely injured, had normal hemoglobin levels on arrival in ED so there was no need for red blood cell transfusion in a lot of patients. In previous studies we have described this phenomenon of normal hemoglobin levels in smaller service areas with short transport times [6, 7].

Interestingly, thromboembolic complications increased over time, though no one died due to thromboembolic complications. Urgent laparotomy was the largest independent predictor (Odds 2.6) for developing thromboembolic complications. The number of urgent laparotomies however did not change over time. Tranexamic acid (TXA) was increasingly used during the study period, so it could have potentially attributed to the increase in thromboembolic complications. However, TXA was not an independent predictor for the development of thromboembolic complications. Several studies have investigated the role of TXA on the development of thrombosis in severely injured patients with varying findings; while some researchers found no significant effect of TXA on thromboembolic complications, others expressed caution or reported a potential increase in thrombotic risk following its use [26–29]. Overall, the thromboembolic complication rate (9%) was fairly high compared to some studies [30, 31], although others have shown similar rates in trauma patients [32]. Since FFP was an independent predictor of thromboembolic complications, its increased use has likely contributed to this rise. In literature, others have also shown that plasma transfusion was associated with an increased risk of early onset of DVT [32]. Further, Zander et al. recommended to be cautious with FFP when early hemodynamic stability could be achieved since FFP was a risk for thromboembolic complications in patients who received less than 4 units of PRBC [33]. Another, recent, study showed that trauma patients requiring more than 3 units of PRBCs who received combined resuscitation with FFP and platelets experienced improved survival and reduced complications with comparable thromboembolic complications [34]. The dilemma of balanced resuscitation in non-exsanguinating patients is still subject to debate [34–36], but these data suggest that the optimal dosage of FFPs should be evaluated in order to find the balance between correcting coagulopathy, restoring blood volume, and managing the inflammatory response after trauma. The ideal dose of FFPs should be determined by the patient ‘s physiology and inflammatory response to the injury and their response to the initial resuscitation. In the future, personalized immunophenotyping may aid in the identification of trauma patients at risk for inflammatory complications [37].

The incidence of infectious complications was around 45%, and did not change over time even though the incidence of pelvic fractures (which was an independent predictor for infections) increased. Prior studies have reported similar infection rates in severely injured populations [15, 38]. Additionally, mortality due to infections was also low. Given the observed decline in MODS- and ARDS-related deaths, both of which are neutrophil-mediated inflammatory conditions, it is plausible that infections represent a residual, milder consequence of reduced systemic inflammation [6].

To our knowledge, this is the first study in which a direct relation has been shown between the change in resuscitation strategies and the development and severity of inflammatory complications in a cohort of severely injured patients. This study has also some limitations. Firstly, it was a retrospective analysis of prospectively collected data and conducted at a single institution, where clinical care and research were managed by the same team, potentially introducing bias. Secondly, routine duplex ultrasound screening for asymptomatic DVT was not performed, meaning that occult, non-clinically relevant thrombosis may have gone undetected. However, prior research by Spain et al. suggested that the clinical significance of DVT detected only through surveillance is low, and the high cost of routine Doppler imaging does not justify its widespread use [39]. Lastly, preexisting medical conditions, which may have influenced patient outcomes were not documented.

In conclusion, resuscitation strategies changed to less crystalloids and more FFP ≤ 24 h in the past decade. With unchanged injury severity, but worse physiological conditions of patients presented over time, this change likely caused a decrease in ARDS and increase in thromboembolic complications. Death due to inflammatory complications was low suggesting a mild course of these inflammatory complications and supporting current resuscitation strategies. However, improved identification of patients who require FFP transfusion may help reduce thromboembolic complications. The future goal should be to determine the optimal FFP dosage to balance the correction of coagulopathy, restoration of blood volume, and management of the inflammatory response following trauma.

## Electronic supplementary material

Below is the link to the electronic supplementary material.

Supplementary Material 1

Supplementary Material 2

Supplementary Material 3

## Data Availability

The dataset supporting the conclusions of this article can be obtained upon reasonable request from the corresponding author.
