# Supplementary material for: Change in resuscitation influenced development and severity of inflammatory complications in severely injured
Source: Eur J Trauma Emerg Surg. 2025 Jun 23;51(1):232. doi: 10.1007/s00068-025-02905-8 (PMC12185553; doi:10.1007/s00068-025-02905-8)
Supplement: Supplementary file 1 — Supplementary Material 1 [file 68_2025_2905_MOESM1_ESM.docx]

**Figure S1.** Flowchart of included patients

**Death <48 h**

n =49

**Total number of trauma patients (age>15) admitted to hospital 2014-2024**

n=11,666

**Patients admitted to ICU, directly or via OR**

n = 2030

**Isolated TBI* (including asphyxiation, drowning, burns)**

n =1396

**Severely injured patients admitted to ICU**

n = 585

* Isolated traumatic brain injury (TBI) was defined as Abbreviated Injury Score (AIS) head >3 and AIS <2 or less in other regions
