# Supplementary material for: Change in resuscitation influenced development and severity of inflammatory complications in severely injured
Source: Eur J Trauma Emerg Surg. 2025 Jun 23;51(1):232. doi: 10.1007/s00068-025-02905-8 (PMC12185553; doi:10.1007/s00068-025-02905-8)
Supplement: Supplementary file 2 — Supplementary Material 2 [file 68_2025_2905_MOESM2_ESM.docx]

**Supplemental Table S1.** Definitions of specific infections.

| Type of infection [reference] | Definition |
| --- | --- |
| Pneumonia [Cole] | Change in character of sputum with increased suction requirements, new onset cough, dyspnea or tachypnea, pyrexia > 38°C, WBC <4,0 or >12,0 x10^9^/L, worsening gaseous exchange, new or progressive infiltrates on chest x-ray study. |
| Fracture related infection [Metsemakers] | Culture of distinct pathogens from at least 2 separate deep tissue/implant specimens in the presence of a fistula, sinus, or wound breakdown (with communication to the bone or implant) and/or purulent drainage from the wound or presence of pus during surgery. clinical signs comprising local redness, swelling, increased local temperature, fever (≥38.3°C), or persistent, increasing, or new-onset wound drainage beyond the first few days. |
| Positive blood culture [Cole] | Recognized pathogen cultured from 1 or more blood cultures or common skin contaminants cultured from 2 or more blood cultures on 2 separate occasions within a 48-hour period. |
| Septic shock [Levy] | Suspected or proven infection and two or more of the following criteria are met: tachycardia (>90/min), tachypnea (>20/min), >38.5°C or temperature < 35.6°C, WBC <4,0 or >12,0 x10^9^/L. associated with persisting organ dysfunction, hypoperfusion, or hypotension despite adequate fluid resuscitation. |
| Abdominal abscess [Horan] | Organisms cultured from purulent material from intraabdominal space obtained during a surgical operation or needle aspiration, or at least 2 of the following signs or symptoms with no other recognized cause: fever (>38°C), nausea, vomiting, abdominal pain, or jaundice and at least 1 of the following:  a. organisms cultured from drainage from surgically placed drain  b. organisms seen on Gram’s stain of drainage or tissue obtained during surgical operation or needle aspiration  c. organisms cultured from blood and radiographic evidence of infection |
| Wound infection [Cole,ACS] | Organisms isolated from wound, discharge or tissue of surgical incision(s) or surgical wound dehiscence and/or visual evidence of surgical site infection diagnosed by a surgeon. Localized heat, redness, swelling, tenderness, pyrexia > 38°C. |
| Thrombophlebitis [Horan] | At least 2 of the following signs or symptoms caused by a superficial venous cannula: pain or tenderness, localized swelling, redness, or heat |
| Thorax empyema [Horan] | Organisms cultured from lung tissue or pleural fluid either during a surgical operation or histopathologic examination or organisms cultured from an abscess cavity seen on radiographic examination of lung. |
| UTI [Cole] | Positive urine culture (collected using appropriate sterile technique) and/or elevated WBC count and inflammatory markers, pyrexia > 38°C, dysuria, abdominal/flank pain. |
| Secondary meningitis [HORAN] | organisms cultured from CSF after craniotomy or pressure monitor insertion or at least 1 of the following signs or symptoms with no other recognized cause: fever (>38°C), headache, stiff neck, meningeal signs, cranial nerve signs, or irritability and at least 1 of the following:  a. increased white cells, elevated protein,and/ or decreased glucose in CSF  b. organisms seen on Gram’s stain of CSF  c. organisms cultured from blood  d. positive antigen test of CSF, blood, or urine  e. diagnostic single antibody titer (IgM) or 4-fold increase in paired sera (IgG) for pathogen |

WBC=white blood cell, CSF= cerebrospinal fluid
